# Intimate Partner Femicide in South Africa in 1999 and 2009

**DOI:** 10.1371/journal.pmed.1001412

**Published:** 2013-04-02

**Authors:** Naeemah Abrahams, Shanaaz Mathews, Lorna J. Martin, Carl Lombard, Rachel Jewkes

**Affiliations:** 1 Gender and Health Research Unit, Medical Research Council, Tygerberg, Cape Town, South Africa; 2 Division of Forensic Medicine, Department of Clinical Laboratory Services, Faculty of Health Sciences, University of Cape, Mowbray, South Africa; 3 Biostatics Unit, Medical Research Council, Tygerberg, Cape Town, South Africa; 4 Gender and Health Research Unit, Medical Research Council, Pretoria, South Africa; University of Ottawa, Canada

## Abstract

**Background:**

Death is the most extreme consequence of intimate partner violence. Female homicide studies with data on the perpetrator–victim relationship can provide insights. We compare the results of two South African national studies of female homicide with similar sampling done 10 y apart.

**Methods and Findings:**

We conducted a retrospective national survey using a weighted cluster design of a proportionate random sample of 38 mortuaries to identify homicides committed in 2009. We abstracted victim data from mortuary and autopsy reports, and perpetrator data from police interviews. We compared homicides of women 14 y and older in 2009 with previously published data collected with the same methodology for homicides committed in 1999.

The study found that the rate of female homicide per 100,000 female population in 2009 was 12.9 (95% confidence interval [CI]: 9.3, 16.5), compared to 24.7 (95% CI: 17.7, 31.6) in 1999. The incidence rate ratio of 0.54 (95% CI: 0.20, 0.84) reflects a significantly lower rate in 2009. The rate of intimate partner femicide was 5.6/100,000 in 2009 versus 8.8/100,000 in 1999, with an incidence rate ratio of 0.63 (95% CI: 0.24, 1.02), indicating no difference between rates. Logistic regression analysis of homicide characteristics showed that the odds ratio of suspected rape among non-intimate femicides in 2009 compared to 1999 was 2.61 (95% CI: 1.23, 4.08) and among intimate partner femicides it was 0.84 (95% CI: 0.50, 1.42). The OR of homicide by gunshot was 0.54 (95% CI: 0.30, 0.99) in 2009 versus 1999. There was a significant drop in convictions of perpetrators of non-intimate femicide in 2009 versus 1999 (OR = 0.32 [95% CI: 0.19, 0.53]). Limitations of the study include the relatively small sample size and having only two time points.

**Conclusions:**

Female homicide in South Africa was lower in 2009 than 1999, but intimate partner femicide and suspected rape homicide rates were not statistically different. The cause of the difference is unknown. The findings suggest that South Africa needs greater efforts nationally to implement evidence-based violence prevention.

*Please see later in the article for the Editors' Summary*

## Abstract 2

Naeemah Abrahams and colleagues compare the incidence of female homicide in women aged over 14 years in South Africa in 1999 and 2009 and analyze the fatal violent attacks perpetrated by intimate partners.

## Editors' Summary

**Background:**

Violence against women (often referred to as gender-based violence) is common, serious, and takes many forms, including physical, sexual, and emotional abuse, and has profound implications for every aspect of women's lives. One of the most common forms of violence is perpetrated by a husband or male partner (often referred to as intimate partner violence), and as it usually happens in private, is often ignored or goes unreported. According to the World Health Organization, population surveys indicate that 10%–69% of women have been abused by an intimate partner. This form of violence is so prevalent because in many countries and cultures, violence against a female partner is often not perceived as a crime but rather as a private family matter.

**Why Was This Study Done?:**

The extreme consequence of violence against women is death, and given the seriousness of the widespread problem of violence against women, there have been many international and national efforts to raise awareness of the issue and to implement policies to reduce such violence. In order for these policies to be most effective, countries implementing strategies to prevent intimate partner violence should also have the capacity to monitor the results of such strategies, but unfortunately, these data are not routinely available. Tracking changes in fatal intimate partner violence (that is, when a woman is killed by an intimate partner, also referred to as intimate femicide) is one possible option of monitoring the impact of policies and programs. So in this study from South Africa, the researchers collected data on and compared the prevalence of intimate femicide at two time points ten years apart (1999 and 2009, between which time points new legislation on gender-based violence was introduced) to examine whether there were any differences.

**What Did the Researchers Do and Find?:**

The researchers analyzed information on female homicide victims, aged 14 years and older, whom they identified from mortuary registers and databases in 2009. The researchers collected cause of death data from the autopsy reports and checked other information via police interview. The researchers then compared these results with a similar study they had conducted for homicides in 1999 but treated each study independently, with a separate statistical analysis, and calculated rates according to the population estimates at each time point.

Using these methods, the researchers found that in 2009, there were 930 female homicides compared to 1,052 in 1999, giving an overall female homicide rate per 100,000 women of 12.9 in 2009 compared to 24.7 in 1999. There was a statistically significant decrease in the rate of non-intimate femicide, with a rate of 8.6 per 100,000 women in 1999 compared to 4.2 in 2009. Although there was some evidence of a decrease in the rate of intimate partner femicide—8.8 per 100,000 women in 1999 compared to 5.6 in 2009—this decrease was not statistically significant. The researchers also found that there was a significant decrease in the rate of fatal shootings (female gun homicides), 7.5 per 100,000 women in 1999 compared to 2.5 in 2009, and that this finding was similar for homicides perpetrated by partners and non-partners (intimate and non-intimate gun homicides). Finally, the researchers found that the overall rate of fatal rapes (female rape homicides) was 3.4 per 100,000 women in 1999 compared to 2.5 in 2009, but again, this difference was not statistically significant. Unfortunately, the researchers found that the odds (chance) of conviction of perpetrators of intimate femicide was unchanged between the two time points (1.11), and the odds of conviction of perpetrators of non-intimate femicides had significantly decreased (0.32).

**What Do These Findings Mean?:**

These findings suggest that, overall, female homicide in South Africa was substantially lower in 2009 than in 1999, but the 2009 figure is still five times the global rate of this crime. The rate of non-intimate femicide declined significantly over the two time points, but there was no statistically significant reduction in intimate femicide. There was a substantial difference in the rate of homicide from gunshot between the two years, most likely explained by gun control legislation. This study has several limitations, including the small number of mortuaries included and the differences in the studies conducted in 1999 and 2009. Nevertheless, this study indicates that a renewed commitment from the South African government is urgently needed to develop policy-driven prevention interventions to reduce female homicide, especially when perpetrated by an intimate partner.

**Additional Information:**

Please access these websites via the online version of this summary at http://dx.doi.org/10.1371/journal.pmed.1001412.

- Violence against Women Online Resources provide lists of sources about violence against women
- The World Health Organization website lists some facts about violence against women
- The US Centers for Disease Control and Prevention provides more information about intimate partner violence
- Sexual Violence Research Initiative provides links to research on sexual violence

## Introduction

The murder of an intimate partner is one of the most extreme consequences of gender-based violence. Although intimate partner violence (IPV) can be perpetrated by both males and females, women are disproportionately killed by their intimate partners [1],[2]. A recent World Health Organization review of risk factors for IPV perpetration showed that risk factors were similar in high income and lower income settings [3]. Men's risk of perpetration starts in childhood and is more common if men have witnessed violence between parents and if they have been exposed to physical and sexual abuse in childhood [3],[4]. Having witnessed or experienced domestic violence in childhood results in more acceptance of violence, lower self-esteem, attachment problems, and personality disorders [3]. Men are more likely to be violent if they have lower income and education and if they abuse substances [3]–[5]. Having multiple partners and greater relationship discord are also risk factors [4],[6],[7]. Gender-based violence is fundamentally rooted in gender inequality. It is more common in communities where there is a cultural emphasis on gender hierarchy, where there is greater acceptability of the use of violence in interpersonal relations, and where men's dominance over and control of women is seen as legitimate [4]. In such communities there are often very weak community sanctions for violence against women. Individual men who view being able to demonstrate control of women as essential to their self-evaluation as men are much more likely to be violent [3]. Women are at greater risk of becoming victims if they themselves accept a subservient position with respect to men (often having learnt it at home by witnessing inter-parental violence), have poor conflict skills, have substance abuse problems, have depression, and are less well educated [3].

In 1998 South Africa adopted the Domestic Violence Act (Act 116 of 1998), promulgated in 1999 [8]. The act provided a framework for protection against and prosecution of a range of offences (physical, sexual, emotional, and economic abuse) by people in a domestic relationship. The act provided for the issuing and enforcement of protection orders and confiscation of weapons from those who had orders against them. In 2007 the state adopted the Criminal Law (Sexual Offences and Related Matters) Amendment Act [9]. This act provided a very broad definition of sexual offences and a strong base from which to tackle the historical near impunity of rape perpetrators. In addition, in 2000 the Firearms Control Act [10] strengthened legal control over hand gun ownership, and this may have had an impact on gun homicides.

Countries implementing strategies to prevent IPV nationally need to have a capacity to monitor these strategies, but monitoring is often not possible using routine information. Analysing trends in fatal IPV (intimate partner femicide) is one means of monitoring overall programming impact, but these data are usually not available routinely. In South Africa we conducted research into intimate partner femicide at two time points 10 y apart to establish whether there were differences. This paper compares the findings for the two time periods on the prevalence and patterns of female homicide and intimate femicide of women aged 14 y and over. A simple brief account of the findings of this study was presented in a report for policy makers in South Africa in 2012 [11].

## Methods

The Ethics Committee of the South African Medical Research Council approved the study, and the national and provincial Departments of Health, the Forensic Pathology Service, and the South African Police Service provided further approval and access to data. The police gave written informed consent before interviews.

We conducted a retrospective, national mortuary-based study of female and child homicide cases that presented at medical legal laboratories between 1 January 2009 and 31 December 2009. This study design was similar to the 1999 national female homicide study [12]. We identified deaths through mortuaries, as according to the Inquest Act of 1959, all unnatural deaths in South Africa undergo a post-mortem examination. We drew a random sample of 38 mortuaries as compared to the 25 in 1999, using proportional allocation from a stratified sampling frame with mortuaries stratified into three groups based on the number of autopsies performed per year. The strata were as follows: small, <500 autopsies per year; medium, 500–1,499 autopsies per year; and large, >1,499 autopsies per year. The sampling fraction for large mortuaries was 55.6% (5/8), for medium-size mortuaries was 39.4% (13/33), and for small mortuaries was 24.7% (20/81). The 1999 study used this stratification to enhance the precision of national estimates, and we repeated it in 2009 for the same reason. We wanted to ensure that the sample was representative and included both small rural mortuaries and large ones attached to medical schools. The restructuring of mortuaries in 2005 resulted in 123 mortuaries operating in 2009 compared to 225 in 1999.

We present an analysis of female homicide victims, aged 14 y and older, identified from mortuary registers and databases. We abstracted data onto a form from autopsy reports, with follow-up interviews with police investigators using a questionnaire to verify the cause of death, identify relationships between the victim and the perpetrator, and to collect other crime investigation data. The data for the 1999 study were collected in 2002–2003, whereas in the 2009 study police interviews were concluded in 2011. The shorter delay in the second study did not appear to adversely affect the availability of data, since there were fewer cases with incomplete data in 2009 than in 1999 (17 cases not traced in the police system in 2009 compared to 147 in 1999).

We collected cause of death data from the autopsy reports and verified the socio-demographic data during the police interview. Autopsy reports provided information about pregnancy and whether rape was suspected. Police verified suspicious rape cases. For both studies, the police provided information on the perpetrator, case outcome, history of IPV, and the relationship of the victim with the perpetrator. We considered intimate partners to include current or former husbands and boyfriends (dating and co-habiting), same-sex sexual partners, and rejected suitors. The identification of rape homicides was also identical in the two studies (see Box 1 for definitions of terms).

**Box 1. Definition of Terms**

**Female homicide**: Killing of women

**Femicide**: Killing of women

**Gender-based homicide**: Homicide with distinct gendered circumstances such as intimate partner femicide and suspected rape homicide

**Intimate femicide/intimate partner femicide**: Killing of women by intimate partners (i.e., a current or former husband/boyfriend, same-sex partner, or rejected would-be lover)

**Non-intimate femicide**: Killing of women by someone other than an intimate partner (stranger, family member, acquaintance, etc.)

**Suspected rape homicide**: Homicide occurring with a sexual component identified during investigation

For the comparison, we considered the 1999 and 2009 surveys as two independent surveys because of the time separation and the independent samples. We applied sampling weights by year and weighted for the total number of mortuaries within the strata. We used the mid-year population estimates from Statistics South Africa for 1999 and 2009 for the calculation of rates. The 1999 rates were based on the population from the 1996 census [13], and the 2009 rates on the 2001 census [14]. These population data, adjusted for undercount and population growth, are used extensively for government and administrative purposes in the country.

All procedures took into account the multi-stage structure of the dataset, with weighting, stratification by mortuary size, and the using mortuaries as clusters. We estimated the homicide rates for all female homicides in 1999 and 2009 and within femicide subgroups (intimate, non-intimate), and 95% confidence limits were calculated using standard methods for estimating confidence intervals from complex multi-stage sample surveys (Taylor linearization). Incidence rate ratios (IRRs) for 2009 compared to 1999 homicide rates were estimated, as well as confidence intervals to facilitate the comparison between years. We did multiple logistic and linear regression analyses to test whether year of survey or type of homicide status was associated with socio-demographic or crime-related variables (proportion of intimate femicide, age of victim, suspected rape, pregnant at time of death, conviction of perpetrator, and mechanism of death [gun injury, stab injury, or blunt injury]). The regression models included interaction effects between the survey year and the type of homicide to evaluate the homogeneity of the year effect across the intimate and non-intimate subgroups.

## Results

All the sampled mortuaries in each year contributed data. Our sample identified 930 female homicides in 2009 compared to 1,052 in 1999. The overall female homicide rate per 100,000 women was 12.9 (95% confidence interval [95% CI]: 9.3, 16.5) in 2009 compared to 24.7 (95% CI: 17.7, 31.6) in 1999, and the estimated IRR was 0.52 (95% CI: 0.20, 0.84), reflecting a significantly lower rate in 2009 (Table 1). A similar statistically significant lower rate of non-intimate femicide was found. The non-intimate femicide rate per 100,000 women was 8.6 (95% CI: 6.2, 11.1) in 1999, compared to 4.2 (95% CI: 3.0, 5.5) in 2009 (IRR: 0.48 [95% CI: 0.18, 0.78]). However, although there was some evidence of a decrease in the rate of intimate femicides per 100,000 women (from 8.8 in 1999 [95% CI: 6.2, 11.2] to 5.6 [95% CI: 4.0, 7.2] in 2009), the decrease was not significant (IRR: 0.63 [95% CI: 0.24, 1.02]).

**Table 1:** Population rates for 1999 and 2009 for all female homicides, intimate femicide, and non-intimate femicide by age, race, gunshot, and suspected rape homicides, and incidence rate ratio of estimates of population rates between study years.

Homicide Characteristic	1999 (Unweighted = 1,052; Weighted = 3,793)			2009 (Unweighted = 930; Weighted = 2,363)			IRR of Population Rate Estimates 2009/1999 (95% CI)
	Female Population	*N* (95% CI)	Rate per 100,000 Population (95% CI)	Female Population	*N* (95% CI)	Rate per 100,000 Population (95% CI)	
**Overall female homicide**	15,360,904	3,793 (2,693, 4,894)	24.7 (17.7, 31.6)	18,273,358	2,363 (1,703, 3,024)	12.9 (9.3, 16.5)	0.52 (0.20, 0.84)
**NIF**	15,360,904	1,335 (959, 1,710)	8.6 (6.2, 11.1)	18,273,358	768 (534, 1,003)	4.2 (3.0, 5.5)	0.48 (0.18, 0.78)
**IF**	15,360,904	1,349 (972, 1,727)	8.8 (6.2, 11.2)	18,273,358	1,024 (725, 1,322)	5.6 (4.0, 7.2)	0.63 (0.24, 1.02)
**IF by age group**							
14–29 y	6,892,855	649 (441, 857)	10.3 (6.3, 12.4)	7,885,758	474 (328, 631)	6.0 (4.2, 8.0)	0.63 (0.25, 1.02)
30–44 y	4,363,286	524 (336, 712)	12.8 (7.7, 16.3)	5,047,200	430 (285, 574)	8.5 (5.6, 11.4)	0.70 (0.23, 1.18)
45–59 y	2,261,298	71 (26, 117)	3.5 (1.1, 5.1)	3,193,500	103 (59, 147)	3.2 (1.8, 4.6)	1.02 (0, 2.08)
60+ y	1,843,465	26 (8, 44)	1.5 (0.4, 2.3)	2,146,900	14 (1, 26)	0.7 (.05, 1.2)	0.46 (0, 1.03)
**IF by victim's racial group**							
African	11,683,651	1,023 (710, 1,336)	8.8 (6.0, 11.4)	14,137,939	801 (563, 1,039)	5.7 (4.0, 7.3)	0.64 (0.24, 1.05)
Coloured	1,375,413	252 (40, 464)	18.3 (2.9, 33.7)	1,711,912	173 (30, 316)	10.1 (1.8, 18.5)	0.55 (0, 1.25)
Indian	424,331	21 (0, 44)	4.9 (0, 10.3)	510,296	18 (0, 35)	3.5 (0, 6.9)	0.71 (0, 1.85)
White	1,974,767	53 (20, 86)	2.8 (1.0, 4.3)	1,912,465	28 (0, 35)	1.5 (0, 1.8)	0.54 (0, 1.15)
**Gunshot homicide**							
All female homicide	15,360,904	1,147 (557, 1,735)	7.5 (3.6, 11.3)	18,273,358	462 (281, 642)	2.5 (1.6, 3.5)	0.33 (0.08, 0.59)
IF	15,360,904	405 (189, 619)	2.7 (1.2, 4.0)	18,273,358	179 (99, 258)	1.0 (0.5, 1.4)	0.37 (0.07, 0.66)
NIF	15,360,904	435 (198, 669)	2.8 (1.3, 4.4)	18,273,358	132 (70, 193)	0.7 (0.4, 1.1)	0.25 (0.04, 0.46)
**Suspected rape homicide**							
All female homicide	15,360,904	526 (246, 806)	3.4 (1.6, 5.2)	18,273,358	455 (306, 605)	2.5 (1.7, 3.3)	0.72 (0.19, 1.25)
IF	15,360,904	151 (63, 239)	1.0 (0.4, 1.6)	18,273,358	108 (64, 151)	0.6 (0.4, 0.8)	0.60 (0.13, 1.07)
NIF	15,360,904	171 (63, 277)	1.1 (0.4, 1.8)	18,273,358	210 (130, 291)	1.2 (0.7, 1.6)	1.03 (0.17, 1.88)

IF, intimate femicide; NIF, non-intimate femicide.

A significantly lower rate of female gun homicides per 100,000 women was found in 2009, with the 1999 rate of 7.5 (95% CI: 3.6, 11.3) much higher than the 2009 rate of 2.5 (95% CI: 1.6, 3.5). The IRR was 0.33 (95% CI: 0.08, 0.59). There was a similar finding for both intimate and non-intimate gun homicides.

The overall female rape homicide rate per 100,000 women for 1999 was 3.4 (95% CI: 1.6, 5.2) compared to 2.5 (95% CI: 1.7, 3.3) for 2009 (Table 1), with an estimated IRR of 0.72 (95% CI: 0.19, 1.25), indicating no difference in the rate of suspected rape homicides for the two years. The IRR of suspected rape homicide by a non-intimate partner between the two studies was 1.03 (95% CI: 0.17, 1.88). Suspected rape homicide by an intimate partner was also not significantly different; the IRR was 0.60 (95% CI: 0.13, 1.07).

A comparison of the characteristics by type of homicide between 2009 and 1999 is shown in Table 2. The overall mean age of the victims did not differ significantly by year of survey. We found a significant age difference of 10.7 y between victims of intimate and non-intimate femicides, and this was consistent over both years. For two categorical characteristics an interaction between the study year and type of femicide was found: suspected rape homicide and whether a perpetrator was convicted. For suspected rape among non-intimate femicides the odds ratio (OR) for year (2009 versus 1999) was 2.61 (95% CI: 1.43, 4.77) (Table 2), but for intimate femicides it was 0.84 (95% CI: 0.50, 1.42), reflecting no year effect. For convictions of perpetrators of non-intimate femicides the OR for year was 0.32 (95% CI: 0.19, 0.53), but for conviction of perpetrators of intimate femicide the OR was 1.11 (95% CI: 0.76, 1.61), reflecting no year effect. Year was not associated with deaths from blunt trauma and sharp injuries, but we found a significant association for gun homicides (OR = 0.54 [95% CI: 0.30, 0.99]), and this association was consistent across the two types of femicides.

**Table 2:** Comparison of homicide characteristics between 1999 and 2009 by type of femicide and effect measure of study year and type of homicide.

Characteristic	Intimate Femicide		Non-Intimate Femicide		Effect Measure of Study Year and Type of Homicide	
	1999 Percent (95% CI)	2009 Percent (95% CI)	1999 Percent (95% CI)	2009 Percent (95% CI)	Year OR (95% CI)	Type of Homicide OR (95% CI)
**Median age (IQR)**	29 (24, 35)	31 (24.3, 37.8)	37 (27, 51)	41 (27.3, 56.3)	1999: 1.00	Non-intimate femicide: 1.00
					2009: 1.83 (−0.29, 3.9)^a^	Intimate femicide: −10.82 (−12.91, −8.73)^a^
**Suspected rape homicide** b	11.4 (7.2, 15.6)	11.0 (7.9, 14.1)	13.2 (7.1, 19.3)	28.5 (21.8, 35.3)	**Intimate femicide**	**1999**
					1999: 1.00	Non-intimate femicide: 1.00
					2009: 0.84 (0.50, 1.42)	Intimate femicide: 0.96 (0.57, 1.61)
					**Non-intimate femicide**	**2009**
					1999: 1.00	Non-intimate femicide: 1.00
					2009: 2.61 (1.43, 4.77)	Intimate femicide: 0.31 (0.20, 0.46)
**Victim pregnant**	2.4 (0.3, 4.5)	3.6 (1.9, 5.3)	1.0 (0.1, 2.3)	3.1 (0.6, 5.6)	1999: 1.00	Non-intimate femicide: 1.00
					2009: 2.17 (0.92, 5.12)	Intimate femicide: 1.30 (0.62, 2.73)
**Perpetrator convicted** b	35.1 (25.4, 44.7)	37.4 (29.2, 45.7)	32.7 (24.5, 41.0)	23.1 (16.9, 29.2)	**Intimate femicide**	**1999**
					1999: 1.00	Non-intimate femicide: 1.00
					2009: 1.11 (0.76, 1.61)	Intimate femicide: 1.10 (0.63, 1.94)
					**Non-intimate femicide**	**2009**
					1999: 1.00	Non-intimate femicide: 1.00
					2009: 0.32 (0.19, 0.53)	Intimate femicide: 3.79 (2.60, 5.52)
**Died from blunt force injuries**	33.2 (24.2, 42.3)	29.5 (23.6, 35.5)	21.2 (14.1, 28.3)	22.4 (16.2, 28.6)	1999: 1.00	Non-intimate femicide: 1.00
					2009: 0.88 (0.58, 1.35)	Intimate femicide: 1.75 (1.29, 2.37)
**Died from gun injuries**	30.6 (19.9, 41.2)	17.4 (11.2, 23.6)	33.6 (23.1, 44.2)	17.1 (10.6, 23.7)	1999: 1.00	Non-intimate femicide: 1.00
					2009: 0.54 (0.30, 0.99)	Intimate femicide: 0.86 (0.53, 1.39)
**Died from stab injuries**	33.2 (25.8, 40.6)	31.4 (25.0, 37.8)	34.3 (23.4, 45.2)	35.5 (28.9, 42.1)	1999: 1.00	Non-intimate femicide: 1.00
					2009: 0.77 (0.50, 1.20)	Intimate femicide: 1.09 (0.82, 1.46)
**Proportion of intimate femicide**	50.2 (44.3, 55.7)	57.1 (51.9, 62.3)				
**Perpetrator committed suicide**	16.6 (10.8, 22.4)	18.2 (13.6, 22.7)				
**History of IPV**	31.6 (22.1, 41.0)	33.0 (26.3, 39.8)				

a Coefficients.

b Characteristics with significant interaction between type of homicide and year.

IQR, interquartile range.

## Discussion

The overall rate of female homicide in South Africa was substantially lower in 2009 than in 1999, and the reasons for this are unknown. The reduction in the overall rate of female homicide found in the study is consistent with the decrease in overall homicides shown in annual police statistics. These show a decrease of 44% between 2003/2004 and 2010/2011 (the police reporting year is 1 April to 31 March) [15]. A statistically significant difference between the years was also found for the rate of non-intimate femicide, but we did not find a statistically significant reduction in the rate of intimate partner femicide. Homicide with suspected rape did not show a parallel decrease.

The lower female homicide rate in 2009 is encouraging, but levels remain high in comparison to other countries. The female homicide rate in 2009 was five times the global rate [16]. The factors driving the decrease overall have not been identified in South Africa, but it appears not to have been changes in the rate of convictions, as the odds of a conviction in cases of intimate femicide was unchanged across the two time periods, whilst that of conviction in non-intimate femicide cases decreased. Our findings similarly do not suggest that the decrease can be explained by a reduction in gender-based homicides, given that we found no significant difference in the intimate femicide rate or the rate of suspected rape homicide. Although the rate of intimate femicide in 2009 was below that found in 1999, at 5.6/100,000 women it was still more than double the rate in the United States (2.0/100,000 women) [17].

We considered whether the rise in the proportion of suspected rape homicides among non-intimate femicide cases could be due to artefact. We consider this unlikely. There is no reason to believe genital examinations have changed since 1999. Rape kits may be used more in autopsies, but this cannot easily explain why we had different findings in autopsies of non-intimate and intimate femicide victims. The nature of the victim–perpetrator relationship is usually not known by the medical examiner at the time of the autopsy. One might have expected that a greater use of rape kits in 2009 would have resulted in more discovery of DNA and a higher rate of perpetrator convictions. This finding was not seen. These findings do not suggest that the increase in the proportion of suspected rape homicides among non-intimate femicide cases is an artefact of improvements in post-mortem examinations.

There was a very substantial difference in the rate of homicide from gunshot between the two years. The decrease is most likely explained by gun control legislation (Firearms Control Act), a policy-driven intervention implemented since 2000 but only fully effective from 2004, with provisions for safer firearm use and ownership amongst its key features [11]. The decrease mirrors findings in high income countries, where female homicide rates have also dropped much more than male homicide rates following reformation of gun laws [18].

In the last decade there have been multiple efforts to improve policing in South Africa [19]. The police force has expanded, the murder case load has been substantially reduced, forensic science laboratories have been strengthened and modernised, and there have been many new initiatives to improve policing and detective work. Yet we found no evidence of improved conviction rates in 2009, and indeed there was a lower likelihood of convictions among the non-intimate cases in 2009 than in 1999. In the 1999 study we showed that identifying a prior history of IPV was very important in securing a conviction [20], and we strongly advocated for the police to put greater efforts into establishing IPV history during the investigation. Ten years later we have found no difference between the two studies in the identification of prior IPV in intimate femicide cases. Research suggests that the proportion of convictions should have increased if police investigated more thoroughly, as it is very infrequent for fatal acts of violence against an intimate partner to be the first instance of partner violence [21],[22]. Furthermore, IPV is very often witnessed in South Africa's overcrowded homes and communities [23]. Rather, it suggests a lack of progress in improving the investigation of female homicide cases and a persisting lack of awareness among police of gender-based motivations for the murder of women.

The research had a number of limitations. The sample size for estimating the population incidence rates in 1999 and 2009 was adequate, but the study lacks power (to detect type 2 error) for the comparison of rates between study years, especially for subgroups. The number of mortuaries that formed the sampling frame was different between the two years, with a smaller sampling frame in 2009. However, we increased our sampling fraction for the middle-sized and smaller mortuary strata, and therefore do not expect our estimates to be affected by the difference in mortuary numbers between the two years. Our findings most likely underestimate the female homicide rate. Our intimate and non-intimate femicide rates were calculated for cases where perpetrators had been identified, and the availability of these data was dependent on information from the police investigation. The proportion of cases missing perpetrator data was not different between the two study years (18.5% in 1999 and 22.9% in 2009, *p* = 0.22), and in neither study year did we have knowledge of bias caused by the missing data [12]. We excluded highly decomposed bodies or female skeletons where cause and mechanism of death could not be established, and numbers were similar across the two years. Such cases are seldom successfully investigated unless a perpetrator reveals the crime. Another limitation is that we have data for only two time points and cannot test for trend in female homicide rates in South Africa. Despite these limitations, our study confirms the value of this model of collecting national intimate femicide data in the absence of a national homicide database. We have also demonstrated that this research method is replicable in resource-limited settings.

This study was conducted in order to investigate whether there were changes in the prevalence and patterns of female homicide in South Africa in 2009 compared to 1999, and we had a particular interest in looking for changes that could have indicated some success of the new gender-based violence legislation and perhaps accompanying prevention programming at a national level. There was evidence of change that we suggest is probably a consequence of gun control legislation, and we did find a difference in female homicide rates overall, but there was a lack of evidence that could be viewed as indicating a positive impact of gender-based violence policies and programming. Whilst we could not rule out type 2 errors, we failed to detect a difference in the non-intimate rape homicide rate, despite a significant reduction in rape homicides overall, and we did not detect a difference in the rate of intimate femicide, despite one being found in the rate of non-intimate femicide. Although the exact factors driving the decrease in female homicide overall are unknown, it does appear that a renewed commitment from government to developing policy-driven prevention interventions is needed to have an impact on the gender-related proportion of female homicide, as well as on the availability of reliable data to monitor trends. The World Health Organization has identified a number of effective evidence-based prevention interventions for gender-based violence [3], and some have been developed in South Africa [24],[25] at both the school and community levels. Globally more research is required to develop an evidence base to support such work.

## Abbreviations

95% CI: 95% confidence interval
IPV: intimate partner violence
IRR: incidence rate ratio
OR: odds ratio
